# Awareness of breast cancer and breast self-examination among female undergraduate students in a higher teachers training college in Cameroon

**DOI:** 10.11604/pamj.2017.28.91.10986

**Published:** 2017-09-29

**Authors:** Carlson-Babila Sama, Bonaventure Dzekem, Jules Kehbila, Cyril Jabea Ekabe, Brice Vofo, Naomi Liteba Abua, Therence Nwana Dingana, Fru Angwafo III

**Affiliations:** 1 Bambalang Sub-Divisional Hospital, Northwest Region, Cameroon; 2 Galactic Corps Research Group (GCRG), Cameroon and Faculty of Health Sciences, University of Buea, Cameroon; 3 Clinical Research Education, Networking and Consultancy (CRENC), Douala, Cameroon and Health Services Partner Cameroon; 4 Grace Community Health and Development Association (GRACHADA), Kumba, Cameroon; 5 Ntam Medicalised Health Centre, Kumba, Cameroon; 6 Catholic General Hospital, Njinikom, Northwest Region, Cameroon; 7 Gynaeco-Obstetric and Paediatric Hospital and Department of Surgery, University Teaching Hospital, Yaoundé, Cameroon

**Keywords:** Breast cancer, breast self-examination, knowledge, practice, female students, Cameroon

## Abstract

**Introduction:**

The incidence of breast cancer (BCa) in Cameroon is on the rise and accounts for a leading cause of mortality. An understanding of the knowledge and practices on breast cancer and breast self-examination (BSE) among teachers are important first steps which will guide the designing of interventions aimed at raising awareness across the general population.

**Methods:**

We conducted a cross-sectional study in April 2016 involving 345 consenting female undergraduate students in the Higher Teachers Training College, Bambili, Cameroon. Data was collected using a pretested self-administered questionnaire and analysed using descriptive methods.

**Results:**

The mean age of the respondents was 22.5±3.2years and a vast majority (n = 304, 88.1%) had heard about BCa primarily from the television/radio (n=196, 64.5%). Overall, less than a quarter (n=65, 21.4%) of respondents who had heard about BCa had sufficient knowledge on its risk factors and signs/symptoms. A plurality (53.3%) thought BCa can be prevented via vaccination while over a third (38.7%) opined that BCa can be treated spiritually. Less than half (47%) of respondents who had heard about BCa had heard about BSE amongst which only 55 (38.5%) had ever practiced it.

**Conclusion:**

Though most students are aware of the existence of breast cancer, their overall knowledge on its risk factors and clinical presentation is insufficient with a concomitant low practice of BSE. These highlighted gaps warrants intensification of sensitization campaigns and educational programmes in order to raise knowledge levels and enhance prevention strategies that would aid in reducing the burden of breast cancer in Cameroon.

## Introduction

Worldwide, breast cancer is the second most frequent cancer and the fifth cause of cancer-related mortality [1]. It is the most common cancer to affect women and it is second only to lung cancer as the principal cause of cancer-related deaths among women [1-3]. In low- and middle-income countries (LMICs), it remains a significant public health challenge as incidence rates have been shown to increase yearly by as much as 5% with over 1 million projected new cases annually by 2020 [2-5]. The emergence of breast disease and subsequent development of cancer appears to be more aggressive in young women compared to its progression in older women [6,7]. In 2008, the prevalence of breast cancer in women ≥15 years in sub-Saharan Africa was estimated at 23.5 per 100,000 women and approximately 35,427 women died from the disease (crude mortality rate of 12.8 per 100.000 women) [8,9]. In Cameroon, the incidence of breast cancer is higher than the worlds average; estimated at 2625 per 100,000 women with a resultant high mortality [3,5]. The high morbidity and mortality due to breast cancer can be in-part reduced if the lesion is detected early enough [2]. In this regard, women need to be “breast aware” by being able to identify the risk factors and symptoms of breast cancer as well as risk reduction strategies.

Though still clouded in controversy, breast self-examination (BSE) still has an important role to play in the early detection of breast cancer in resource-constraint settings where routine clinical breast examination and mammography may not be feasible. In such settings, BSE is recommended because it is free, private, painless, easy, safe, and requires no specific equipment. It has also been shown to improve breast health awareness and thus potentially allow for early detection of breast anomalies [10-13]. The American Cancer Society also recommends that women from the age of 20 years onwards should be educated on the benefits of performing BSE monthly [14]. It had been demonstrated that factors related to women´s awareness, knowledge and perceptions about breast cancer may contribute significantly to medical help-seeking behaviours [15-17]. Thus, considering the potential pivotal role played by teachers in information dissemination, this study sought to assess the awareness, knowledge and perceptions of breast cancer and practice of breast self-examination among female undergraduate students in a higher institution of teaching as this will be essential in informing policy for targeted interventions through the provision of guided educational training programs.

## Methods

**Study design, setting and participants:** We conducted a descriptive cross-sectional study on the 11th of April 2016 at the Higher Teachers Training College (HTTC) Bambili, University of Bamenda in the Northwest Region of Cameroon. Bambili is a centre of attraction for a youthful multi-ethnic population who either move there for studies or to explore the diverse economic activities triggered by the presence of the university. The undergraduate program in HTTC is a three year course and annually, this college graduates about 500 trained teachers. The target population was first cycle female undergraduate students. To consider equal chances of participation, the students were informed about a free and voluntary participation in a breast cancer survey 1 week earlier via oral message during lecture hours. On the said day of the survey, students were consecutively approached in their respective lecture halls for inclusion.

**Study procedures and data collection:** A structured and self-administered questionnaire was developed by the researchers after an extensive review of literature [10-12,18]. The validity of its contents was established through consultation with experts and was pretested on 41 first cycle students from HTTC who were eventually restrained from participating in the final study. Other than concerns about some ambiguous words which were simplified in the revised version, all pilot students reported they easily understood the questionnaire. It had three sections: socio-demographic characteristics, knowledge about breast cancer, and a section on BSE. Data collection facilitators (1 student per academic level) underwent a 2-hour training one day prior to the census. Coding of questionnaires rather than using names was done in order to ensure confidentiality. Consenting participants were handed printed copies of the questionnaire and allowed time to fill their responses and return them anonymously to the facilitators. The completeness of returned questionnaires was visually checked on a daily basis by the principal investigators. The study was approved by the ethics committee of the regional delegation of public health for the Northwest Region and all recruited students signed a consent form.

**Scoring of knowledge:** Each of the questions on knowledge of risk factors and clinical presentation (signs/symptoms) of breast cancer was equitably scored. Categorical responses (Yes/No/Don't know) were applied for the question items. We assigned one point (1) to a correct answer and zero (0) for don't know or an incorrect answer. The overall knowledge score was calculated by summing scores of all knowledge questions (16 on risk factors and 12 on signs/symptoms) yielding a possible range of overall scores from 0 to 28. Scores were divided into two categories: insufficient knowledge (< 50% of correct answers) and sufficient knowledge (≥ 50% of correct answers).

**Statistical analysis:** Data from the questionnaires were entered and analysed using statistical package for the social sciences (SPSS Inc., Chicago, IL) version 20.0. We summarised continuous variables as means and standard deviations (SD), and categorical variables as count and percentages.

## Results

**Socio-demographic characteristics :** Of the 420 questionnaires distributed, 391 (93.1%) were returned amongst which 345 were properly filled, thus subjected to analysis. The participants were between 17 and 34 years (mean = 22.5 ± 3.2) of age. Half (49.9%) of them were in the age range 21-25 years. A vast majority (90.7%) were Christians while almost two-thirds (64.6%) of the respondents were in their first year of studies. Sixty-two (18%) were married (Table 1).

**Table 1:** Socio-demographic characteristics of the included 345 female undergraduates from HTTC, University of Bamenda, Cameroon, April 2016

Variables	Frequency	Percentage (%)
**Age**		
≤20	111	32.2
21 – 25	172	49.9
26 – 30	60	17.4
> 30	2	0.6
**Religion**		
Christian	313	90.7
Muslim	24	6.9
Others	8	2.3
Marital status		
Married	62	18.0
Single	283	82.0
**Academic level**		
1^st^ year	223	64.6
2^nd^ year	95	27.5
3^rd^ year	27	7.9

**Figure 1:**
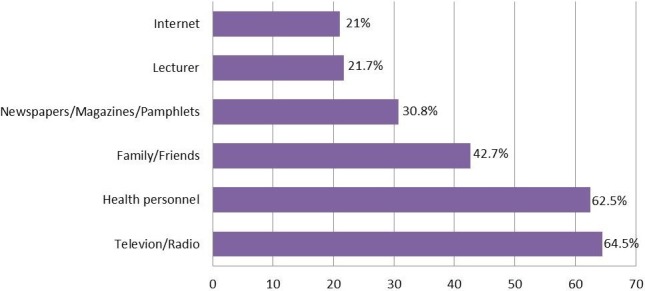
Distribution of participants according to sources of knowledge about breast cancer

**Awareness, knowledge and attitudes on breast cancer:** Of the 345 participants, 41 (11.9%) reported to have never heard about breast cancer. Television/radio (n =196, 64.5%) and health personnel (n =190, 62.5%) were the main sources of knowledge for the 304 (88.1%) participants who had heard about breast cancer (Figure 1). Further analysis will include only the 304 participants who knew what breast cancer was. Of these, 18 (5.9%) had a family history of breast cancer and about one fifth (21.1%) of these female students responded “No” to the question item “will you allow a male doctor to examine your breast” Table 2 summarizes the perceptions towards breast cancer among the 304 participants. Exposure to radiation (n =179, 58.9%), hormone replacement therapy (n = 177, 58.2%), smoking (n = 177, 58.2%), alcohol consumption (n = 142, 46.7%) and high fat diet (n = 138, 45.4%) were the most frequently indexed risk factors for breast cancer. Meanwhile, late menopause (n = 28, 9.2%), early age at first menstruation (n = 25, 8.2%) and not having a child (n = 24, 7.9%) were the least recognized risk factors. Witchcraft was implicated as a potential cause of breast cancer by a third (n =106, 34.9%) of the respondents. The most common symptom of breast cancer identified was lump in the breast (n =248, 81.6%) while over 77% (n =235) of the respondents did not know that lump under the armpit could be a sign of breast cancer.

**Table 2:** Frequency distribution of knowledge and misperceptions towards breast cancer in 304 female undergraduates from HTTC, University of Bamenda, Cameroon, April 2016

Variables	Frequency	Percentage (%)
**Perceived causes/risk factors for developing breast cancer**		
Gender	33	10.9
Increasing age	51	16.8
Positive family history	103	33.9
High fat diet	138	45.4
Smoking	177	58.2
Race/ethnicity	97	31.9
Exposure to radiation	179	58.9
Alcohol consumption	142	46.7
First child at late age	66	21.7
Early age at first menstruation	25	8.2
Late menopause	28	9.2
Inactivity and sedentary lifestyle	122	40.1
Obesity	108	35.5
Hormone replacement therapy	177	58.2
Personal history of breast cancer	84	27.6
Not having a child	24	7.9
Witchcraft&	106	34.9
Wearing tight brassiere&	54	17.8
**Perceived signs/symptoms related to breast cancer**		
Lump in the breast	248	81.6
Discharge from the breast	166	54.6
Pain or soreness in the breast	193	63.5
Change in the size of the breast	174	57.2
Discoloration/dimpling of the breast	104	34.2
Ulceration of the breast	111	36.5
Weight loss	64	21.1
Changes in the shape of the breast	195	64.1
Inversion/pulling in of nipple	79	26.0
Swelling or enlargement of the breast	155	51.0
Lump under armpit	69	22.7
Scaling/dry skin in nipple region	81	26.6

& Wrong answer

The mean knowledge score on risk factors was 5.2 ± 2.7 (range: 0-14). Only 32 (10.5%) of respondents had sufficient knowledge of risk factors of breast cancer. The mean score for knowledge on signs/symptoms of breast cancer was 5.4 ± 3.1 ranging from 0 to 12. One hundred and eleven (36.5%) had sufficient knowledge on the signs/symptoms of breast cancer. The overall knowledge score ranged from 0-23 with a mean score of 10.7 ± 4.8. Overall, less than a quarter (n = 65, 21.4%) of participants had sufficient knowledge on breast cancer. With respect to their attitudes if diagnosed with breast cancer, almost half (n =145, 47.7%) said they will go to a prayer house, 58 (19.1%) will use traditional medicine and only 105 (34.5%) will agree to perform mastectomy if necessary (Table 3). Of the 304 participants who had heard about breast cancer, 287 (94.4%) agreed that breast cancer could be prevented amongst which breast examination was the commonest mode of prevention cited (n =237, 82.6%). Over half (n =153, 53.3%), 104 (36.2%) also thought breast cancer could be prevented by vaccination and physical exercise respectively (Table 4). A minority (n =35, 11.5%) did not know that breast cancer could be treated. Of those who knew (n =269, 88.5%), 248 (92.2%) and 104 (38.7%) said it could be treated medically and spiritually respectively (Table 4).

**Table 3:** Perceived attitudes regarding development of breast cancer of 304 female undergraduates from HTTC, University of Bamenda, Cameroon, April 2016

Variable	Frequency	Percentage (%)
Will be scared	242	79.6
Will consult to a doctor	273	89.8
Will use traditional medicine	58	19.1
Will go to a prayer house	145	47.7
Will agree to perform mastectomy if necessary (removal of the breast)	105	34.5

**Table 4:** Perceptions about prevention and treatment options of breast cancer among female undergraduates from HTTC, University of Bamenda, Cameroon, April 2016

Perceived methods of preventing breast cancer (*n* = 287)		
Variables	Frequency	Percentage (%)
Dieting	92	32.1
Physical exercise	104	36.2
Vaccination	153	53.3
Breast examination	237	82.6
Sucking of breast by male partner	37	12.9
**Perceived methods of treating breast cancer (*n* = 269)**		
**Variables**	**Frequency**	**Percentage (%)**
Medically	248	92.2
Spiritually	104	38.7
Traditionally	47	17.5

**Figure 2:**
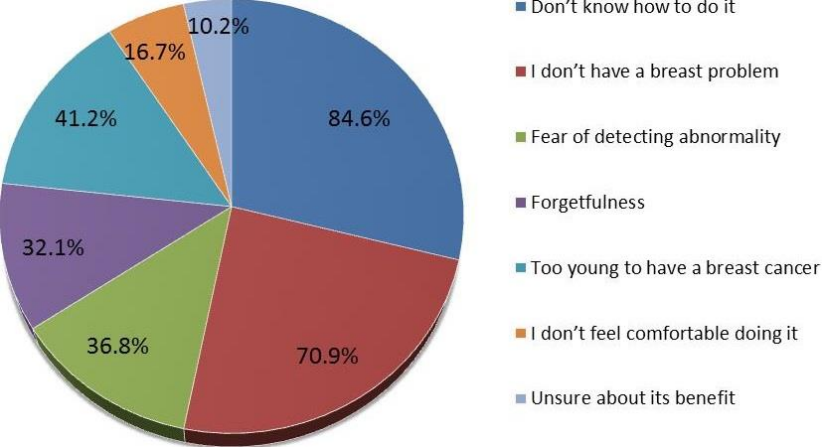
Reasons for not performing breast-self examination

**Awareness and practice of breast self-examination:** Less than half (n =143, 47%) of those who knew about breast cancer had heard about breast self-examination (BSE). Majority (n = 47, 32.9%) did not know how often BSE should be performed while only a quarter (n =37, 25.9%) correctly stated that it should be performed monthly (Table 5). As little as 10 (7%) participants knew that the appropriate time to perform a BSE was few days after menstruation. Despite a substantial proportion (n =88, 61.5%) of students who had never performed BSE, most (n =133, 93%) recognised the importance of BSE for their health. Reasons for not performing BSE are summarised in Figure 2. Of the 95 participants that responded to the question item on the appropriate age to commence BSE, only 3 (3.2%) correctly stated that 20 years was the appropriate age to commence BSE.

**Table 5:** Knowledge, practice and perceived importance of breast self-examination among female undergraduates from HTTC, University of Bamenda, Cameroon, April 2016

Knowledge/Practice	Response	Frequency	Percentage (%)
**Ever heard about BSE**			
	Yes	143	47.0
	No	164	53.0
**Frequency of BSE**			
	Daily	14	9.8
	Weekly	14	9.8
	Monthly&	37	25.9
	Every 3 months	18	12.9
	Every 6 months	8	5.6
	Yearly	5	3.5
	Don’t Know	47	32.9
**Appropriate time of BSE**			
	Few days before menses	7	4.9
	Few days after menses&	10	7.0
	During menses	8	5.6
	No specific time	48	33.6
	Don’t know	70	49.0
**Impression on importance of BSE**			
	Important	133	93.0
	Not important	10	7.0
**Ever performed BSE**			
	Yes	55	38.5
	No	88	61.5
**Previous training on BSE**			
	Yes	25	17.5
	No	118	82.5
**Desire training on how to do BSE**			
	Yes	136	94.4
	No	8	5.6

& Correct answers

## Discussion

Our findings have shown considerable awareness about the existence of breast cancer, but insufficient knowledge and misperceptions on its risk factors and causes as well as infrequent practice of breast self-examination. In this study, 88.1% of participants had heard about breast cancer. This is higher than the 81.2% and 64% observed in a group of Malaysian [19] and Iranian [20] women respectively. It is however much lower than the 100% among female medical students in Harar, Ethiopia [6], 98.7% among female students in the University of Ibadan, Nigeria [21] and 95% among female university students in Ghana [22]. The lower rate of awareness on the existence of breast cancer in our study with respect to these studies may be due to the fact that breast cancer is part of a medical curriculum while it has been adopted in the curricula in the other two universities in a bid to raise awareness. Though about 9 in 10 of our participants knew about breast cancer, our findings reveal a poor understanding and misperceptions on its risk factors, signs/symptoms, prevention and treatment. More than two-thirds of the respondents did not identify gender, increasing age, race/ethnicity, and positive family history, first child at late age, early menarche, late menopause, positive personal history, and nulliparity as potential risk factors of breast cancer. Knowledge gaps about risk factors has also been reported elsewhere among the general population [19,23], university students in Angola [24], female medical students in Saudi Arabia [25], nurses in Pakistan [26] and female teachers in Malaysia [27] and Kuwait [28]. With regards to misperceptions, 17.8% and 34.9% of participants cited wearing of tight brassieres and witchcraft respectively as risk factors of breast cancer. This is in line with a community survey in semi-urban Cameroon [11], studies on rural women [29] and market women [16] in Ibadan, Nigeria and female medical students in Ethiopia [6] that suggests women still attribute the occurrence of breast cancer to a mystical origin. Among others, they considered it “a spiritual attack”, “God's curse”, and “attack from the enemy”. This observation was not that different from reports in a more developed setting; female teachers in Saudi Arabia attributed the occurrence of breast cancer to God and belief in the evil eye [30] while 96.8% of Arab-speaking women in Qatar attributed its occurrence to fate/destiny and less than one-fifth to Gods' punishment and bad luck [31]. As observed elsewhere [6,18,28,32] the commonest symptom of breast cancer identified by our respondents was breast lump. However, knowledge about other signs/symptoms was unsatisfactory. Thus, the need for further health education on the risk factors and clinical presentation of breast cancer is desirable.

Most (94.4%) of our participants perceived that breast cancer could be prevented which is similar to the 95% reported by Suh et al [11]. Though breast examination was mentioned by the majority (82.6%) as a preventive method, a plurality (53.3%) of them incorrectly mentioned vaccination against breast cancer as a preventive method. Interestingly, 12.9% of these respondents also identified sucking of the breast by a male partner as a method of preventing breast cancer. This idea may have emanated, in part, from a recent social media circular within the study setting which suggested girls should make their breast more available to a male partner as regular sucking will help in reducing their risk of developing breast cancer. This calls for urgent actions by authorities to foster awareness on breast cancer in university milieus as such an act has not been proven to prevent breast cancer, and may also promote sexual immorality; thus increasing the risk of acquiring sexually transmitted infections including HIV/AIDS as well as unwanted pregnancies. Though a small proportion, it however remains disturbing that 11.5% of our respondents agreed that breast cancer cannot be treated. This observation concurs to that of Oluwatosin et al [33] in the Akinyele locality in rural Nigeria where the women had even attributed a local name for breast cancer; “jejere” which means “that which devours”. Oladimeji and colleagues [16] also noted that 30% of women in their study considered breast cancer a fast killer. These may be an indication that the myth suggesting that “breast cancer equals death” is still deeply rooted across communities. These wrong assumptions may partly account for some of the reasons why patients present late to hospitals with advanced disease states. Other than seldom mass campaigns for breast health awareness and screening organized by the ministry of public health, there is no national screening program for breast cancer in Cameroon. Introduction of such a program will greatly aid in increasing awareness, eliminate mythical concerns as well as lead to early detection of breast anomalies, hence better prognosis. In a 20-year retrospective analysis of the profiles of 531 breast cancer patients followed up at the Yaoundé General Hospital, Cameroon, Ngowa and collaborators [34] noted that there was a mean delay of 10.35 months with some taking up to 52 months between the apparition of the first signs of breast cancer and presentation for first medical evaluation. As a consequence of this late presentation, none of the patients presented with carcinoma in situ. More than half of them had solicited traditional treatment and visited spiritual houses at first intention before their first medical evaluation. Lack of awareness on breast cancer, ignorance, cultural beliefs and the fear of mastectomy as a treatment modality in hospitals were major setbacks to early presentation. These observations equally concurred to findings in this study with regards to the attitudes of our participants if they developed breast cancer.

Early detection of breast cancer plays a pivotal role in reducing related mortalities. Until circumstances are favourable for routine mammographic screening in resource-limited settings, emphasis should be oriented towards encouraging women to regularly practice BSE. Freeman and collaborators [35] had reiterated the need for young girls to be properly taught BSE as this will greatly influence their practice as they grow older. Though controversies still exists over its effectiveness in reducing mortality [36], the technique remains an important tool for early detection especially in low- and middle-income countries where access to diagnostic and curative facilities may be problematic [4,10,12,37]. In this study, only 38.5% of the participants had ever performed a BSE. It is comparable to the 41% reported by Nde et al [10] among female undergraduate students in the University of Buea, Cameroon, the 42.6% among female undergraduate students in Kirkuk University, Iraq [38], the 37.3% among health extension workers in Ethiopia [12] and 29% in Senegal [39]. Similarly, though close to three-quarters of female undergraduate students in the Ahmadu Bello University, Zaria, Nigeria had heard about BSE, only about one in five had ever practiced it [40]. Our findings suggests the need to increase awareness of BSE as a screening tool as it renders women more “breast aware” and thus potentially allow for early detection of breast cancer. In so doing, various breast-conserving procedures including lumpectomy, segmentectomy, and quadrantectomy may be warranted in patients with early stage cancers rather than the generally feared mastectomy. Raising awareness on the possibilities of breast reconstruction surgery is also warranted as this may improve medical help seeking attitudes of sufferers.

**Limitations:** Other than the lack of a statistical sample estimate, our findings are confined to a group of young educated women which does not necessarily reflect the situation among women in rural areas, thus a potential limitation. Furthermore, this study was conducted in a single department in the university, thus, may not portray the full picture of awareness/perceptions of breast cancer and practice of BSE among female students in the entire university and other state/private universities within the country. Also, the students were not assessed on their ability to correctly perform BSE.

## Conclusion

Female undergraduate students in the Higher Teachers Training College Bambili have insufficient knowledge on breast cancer with poor practice of BSE. Massive health education campaigns designed to enlighten not only female university students in this setting, but also the public at large on the potential causes, risk factors, signs/symptoms, prevention and treatment of breast cancer should be promoted. The unique role of mass media, particularly television/radio to reach a large audience at the same time should be fully explored in order to provide comprehensive information about breast cancer. These breast awareness campaigns should also seek to dispel spirituality and myths regarding the occurrence of breast cancer. Taking into consideration the invaluable role that can be played by BSE in such a resource-disadvantaged setting with a concomitant high burden of breast cancer, there is an urgent need for focused strategies to implement and re-enforce existing cancer awareness and the potential benefits breast self-examination.

### What is known about this topic

- Generally, Cameroonian female students have poor knowledge on breast cancer and infrequently practice breast-self-examination.

### What this study adds

- Narrows awareness, knowledge levels and practice to a group of undergraduate teachers;
- Breast lump is the most commonly known symptom of breast cancer: important knowledge deficits on signs/symptoms and treatment of breast cancer were noted;
- These students have poor attitudes, misperceptions and myths regarding breast cancer; many will avert mastectomy, majority will seek spiritual/traditional healing.

## Competing interests

The authors declare no competing interest.

## References

[1] Ferlay J, Shin H-R, Bray F, Forman D, Mathers C, Parkin D (2010). Estimates of worldwide burden of cancer in 2008: GLOBOCAN 2008. Int J Cancer..

[2] World Health Organisation (2013). Breast cancer: prevention and control..

[3] Ferlay J, Soerjomataram I, Dikshit R, Eser S, Mathers C, Rebelo M (2015). Cancer incidence and mortality worldwide: sources, methods and major patterns in GLOBOCAN 2012. Int J Cancer..

[4] Anderson B, Shyyan R, Eniu A, Smith R, Yip C, Bese N (2006). Breast cancer in limited-resource countries: an overview of the Breast Health Global Initiative 2005 guidelines. Breast J..

[5] IARC (2012). Globocan 2012: estimated cancer incidence, mortality and prevalence worldwide in 2012.

[6] Ameer K, Abdulie S, Pal S, Arebo K, Kassa G (2014). Breast Cancer Awareness and Practice of Breast Self-Examination among Female Medical Students in Haramaya University, Harar, Ethiopia. IJIMS..

[7] Anders C, Hsu D, Broadwater G, Acharya C, Foekens J (2008). Young age at diagnosis correlates with worse prognosis and defines a subset of breast cancers with shared patterns of gene expression. J Clin Oncol..

[8] Bray F, Ren J-S, Masuyer E, Ferlay J (2013). Global estimates of cancer prevalence for 27 sites in the adult population in 200. Int J Cancer.

[9] IARC (2008). Globocan 2008: Cancer Incidence, Mortality and Prevalence Worldwide in 2008.

[10] Nde F, Assob J, Kwenti T, Njunda A, Tainenbe T (2015). Knowledge, attitude and practice of breast self-examination among female undergraduate students in the University of Buea. BMC Research Notes..

[11] Suh M, Atashili J, Fuh E, Eta V (2012). Breast Self-Examination and breast cancer awareness in women in developing countries: a survey of women in Buea, Cameroon. BMC Research Notes..

[12] Azage M, Abeje G, Mekonnen A (2013). Assessment of Factors Associated with Breast Self-Examination among Health Extension Workers in West Gojjam Zone, Northwest Ethiopia. International Journal of Breast Cancer..

[13] Ginseng G, Lauer J, Zelle S, Baeten S, Baltussen R (2012). Cost effectiveness of strategies to combat breast, cervical, and colorectal cancer in Sub-Saharan Africa and South East Asia: mathemetical modelling study. BMJ..

[14] The American Cancer Society (2014). Breast Cancer Prevention and Early Detection.

[15] Okobia M, Bunker C, Okonofua F, Osime U (2006). Knowledge, attitude and practice of Nigerian women towards breast cancer: a cross-sectional study. World J Surg Oncol..

[16] Oladimeji K, Tsoka-Gwegweni J, Igbodekwe F, Twomey M, Akolo C, Balarabe H (2015). Knowledge and Beliefs of Breast Self- Examination and Breast Cancer among Market Women in Ibadan, South West, Nigeria. PLoS ONE..

[17] Hadi M, Hassali M, Shafie A, Awaisu A (2010). Evaluation of breast cancer awareness among female University students in Malaysia. Pharm Pract (Internet).

[18] Lemlem S, Sinishaw W, Hailu M, Abebe M, Aregay A (2013). Assessment of Knowledge of Breast Cancer and Screening Methods among Nurses in University Hospitals in Addis Ababa, Ethiopia, 2011. ISRN Oncology.

[19] Al-Dubai S, Qureshi A, Saif-Ali R, Ganasegeran k, Alwan m, Hadi j (2011). Awareness and Knowledge of Breast Cancer and Mammography among a Group of Malaysian Women in Shah Alam. Asian Pacific J Cancer Prev..

[20] Montazeri A, Vahdaninia M, Harirchi I (2008). Breast cancer in Iran: Need for greater women awareness of warning signs and effective screening methods. Asia Pac Family Med..

[21] Chioma C, Asuzu S (2007). Knowledge, attitude and practice of self-breast examination among the female students of the University of Ibadan, Nigeria. Pakistan J Social Sci..

[22] Sarfo L, Dorothy A, Elizabeth A, Florence A (2013). Knowledge, attitude and practice of self-breast examination among female university students at Presbyterian University College, Ghana. Am J Res Communication.

[23] Amin T, Mulhim A, Al Meqihwi A (2009). Breast cancer knowledge, risk factors and screening among adult Saudi women in a primary health care setting. Asian Pacific J Cancer Prev..

[24] Sambanje M, Mafuvadze B (2012). Breast cancer knowledge and awareness among university students in Angola. Pan Afr Med J..

[25] Nemenqani D, Abdelmaqsoud S, Al-Malki A, Oraija A, Al-Otaibi E (2014). Knowledge, attitude and practice of breast self examination and breast cancer among female medical students in Taif, Saudi Arabia. Open Journal of Preventive Medicine.

[26] Ahmed F, Mahmud S, Hatcher J (2006). Breast cancer risk factor knowledge among nurses in teaching hospitals of Karachi, Pakistan: a cross-sectional study. BMC Nursing.

[27] Parsa P, Kandiah M, Mohd Zulkefli N (2008). Knowledge and behavior regarding breast cancer screening among female teachers in Selangor, Malaysia. Asian Pac J Cancer Prev..

[28] Alharbi N, Alshammari M, Almutairi B, Makboul G, El-Shazly M (2012). Knowledge, awareness, and practices concerning breast cancer among Kuwaiti female school teachers. Alexandria Journal of Medicine..

[29] Oluwatosin O (2006). Rural women's perception of breast cancer and its earlydetection measures in Ibadan, Nigeria. Cancer Nurs..

[30] Dandash K, Al-Mohaimeed A (2007). Knowledge, Attitudes, and Practices Surrounding Breast Cancer and Screening in Female Teachers of Buraidah, Saudi Arabia. International Journal of Health Sciences, Qassim University..

[31] Donnelly T, Khater A, Al-Bader S, Kuwari M, Al-Meer N, Malik M (2013). Beliefs and attitudes about breast cancer and screening practices among Arab women living in Qatar: a cross-sectional study. BMC Women’s Health.

[32] Sim H, Seah M, Tan S (2009). Breast cancer knowledge and screening practices: a survey of 1,000 Asian women. Singapore Med J..

[33] Oluwatosin O, Oladepo O (2006). Knowledge of breast cancer and its early detection measures among rural women in Akinyele Local Government Area, Ibadan, Nigeria. BMC Cancer.

[34] Kemfang Ngowa J, Yomi J, Kasia J, Mawamba Y, Ekortarh A, Vlastos G (2011). Breast Cancer Profile in a Group of Patients Followed up at the Radiation Therapy Unit of the Yaounde General Hospital, Cameroon. Obstetrics and Gynecology International.

[35] Freeman A, Scott c, Waxman A, Arcona S (2002). What do adolescent females know about breast cancer and prevention?. Pediatr Adolesc Gynecol..

[36] Thomas D, Gao D, Ray R (2002). Randomized trial of breast self-examination in Shanghai: final results. J National Cancer Institute..

[37] Anderson B (2007). Guideline implementation for breast healthcare in low-income and middle income countries: overview of the Breast Health Global Initiative Global Summit. Cancer.

[38] Alwan N, Al-Diwan J, Al-Attar W, Eliessa R (2012). Knowledge, attitude & practice towards breast cancer & breast self examination in Kirkuk University, Iraq. Asian Pacific Journal of Reproduction.

[39] Gueye S, Bawa K, Ba M, Mendes V, Toure C, Moreau J (2009). Breast cancer screening in Dakar: knowledge and practice of breast self examination among a female population in Senegal. Rev Med Brux..

[40] Gwarzo U, Sabitu K, Idris S (2009). Knowledge and practice of breast-self examination among female undergraduate students of Ahmadu Bello University Zaria, northwestern Nigeria. Ann Afr Med..

